# Performance evaluation of vacuum blood collection tubes in multicenter clinical laboratories: a real-world study from Southwest China

**DOI:** 10.3389/fpubh.2026.1895353

**Published:** 2026-09-10

**Authors:** Changrui Sun, Chunbao Xie, Xuelong Xie, Li Li, Jian Xu, Jing Ren, Dan Zheng, Juan Du, Zaixiang Xie, Huan Ding, Lan Huang, Bing Zhang, Yi Huang

**Affiliations:** 1 Department of Laboratory Medicine, Sichuan Academy of Medical Sciences and Sichuan Provincial People’s Hospital, Chengdu, China; 2 Department of Clinical Laboratory, The Second People’s Hospital of Yibin·West China Yibin Hospital, Sichuan University, Yibin, China; 3 Department of Clinical Laboratory, Chinese and Western Medicine Hospital of Panzhihua, Panzhihua, China; 4 Department of Medical Laboratory, Dazhou Central Hospital, Dazhou, China; 5 Department of Hospital Laboratory, Guangyuan Central Hospital, Guangyuan, Sichuan, China; 6 Center of Clinical Laboratory Medicine, Guanghan People's Hospital, Deyang, Sichuan, China; 7 Department of Laboratory Medicine, People's Hospital of Ganzi Tibetan Autonomous Prefecture, Kangding, Sichuan, China; 8 Department of Clinical Laboratory, Qianjiang District Hospital of Traditional Chinese Medicine, Chongqing, China; 9 Department of Clinical Laboratory, Zhongxian People's Hospital of Chongqing, Chongqing, China; 10 Department of Laboratory Medicine, Nanchong Central Hospital, Nanchong, Sichuan, China

**Keywords:** multicenter study, performance evaluation, real-world study, Southwest China, vacuum blood collection tubes

## Abstract

**Background:**

Pre-analytical errors account for 60–70% of total laboratory errors, with vacuum blood collection tube quality being a key contributor. Southwest China’s complex terrain (plain/plateau areas) and demand for inter-hospital test result mutual recognition highlight the need for region-specific performance data.

**Methods:**

This multicenter real-world study evaluated 10 blood collection tube brands across 10 hospitals (plain/plateau areas) in Sichuan-Chongqing. Nine physical performance indicators (appearance, tube strength, fill volume, stopper shedding, hemolysis, fibrin adhesion, separation gel integrity, anticoagulation efficacy, and tube-body integrity) were verified against BD tubes, following CLSI GP34-A2 and WS/T 224–2018, with additional clinical consistency analyses for hematological, coagulation, and biochemical parameters. Statistical analysis included linear regression (*R*^2^) and relative/comparison bias.

**Results:**

Widespread performance deficiencies were observed. At the hospital level, fill-volume non-compliance affected 78.3% (7/9) of plain-area hospitals, while at the tube-batch level, 20.0% (1/5) of batches failed at the single plateau site. For stopper shedding, 83.3% (25/30) of tube batches across all sites exceeded the acceptable threshold, corresponding to 80% (8/10) of hospitals having at least one non-compliant tube type. Tube appearance, structural strength, and separation gel integrity were universally compliant across all sites.

**Conclusion:**

Southwest China’s routinely used vacuum blood collection tubes exhibit critical performance gaps, particularly in fill-volume accuracy and stopper integrity. Routine verification of high-risk indicators, site-adaptive batch-specific verification for high-altitude facilities, and manufacturing improvements (e.g., stopper material optimization) are recommended. This study provides quality benchmarks for regional test result mutual recognition and pre-analytical quality improvement.

## 1 Introduction

Vacuum blood collection tubes serve as indispensable tools in clinical laboratory diagnostics, undertaking two core functions: standardized quantitative blood sampling to ensure the consistency of test specimens, and closed-system blood collection that minimizes the risk of nosocomial infections and occupational exposure for medical staff. Notably, pre-analytical errors account for 60–70% of total laboratory errors (as documented in multiple Clinical and Laboratory Standards Institute (CLSI) guidelines and epidemiological studies on laboratory medicine quality), and the quality of vacuum blood collection tubes has been identified as a key and underrecognized contributor to such errors (1, 2). Defects in collection tubes, such as stopper shedding during puncture, or inadequate anticoagulant performance, can trigger a cascade of adverse clinical outcomes: repeated blood draws that compromise patient experience and increase the risk of anemia in vulnerable populations, biased test results that mislead clinical decision-making (e.g., erroneous coagulation parameters causing inappropriate anticoagulant therapy), and unnecessary waste of medical resources including labor, reagents, and diagnostic equipment (3–7).

Southwest China, encompassing Sichuan and Chongqing, presents unique challenges for diagnostic specimen collection: its complex terrain includes high-altitude areas and a wide spectrum of hospital tiers ranging from tertiary grade A hospitals to county-level secondary hospitals, leading to heterogeneous usage scenarios of collection tubes. Against the backdrop of the ongoing initiative for mutual recognition of test results among clinical laboratories in Sichuan-Chongqing region, establishing unified performance standards for vacuum blood collection tubes has become an urgent clinical demand to ensure result comparability across institutions. Existing research on vacuum blood collection tube validation is predominantly limited to single-center studies conducted under ideal laboratory conditions, lacking real-world multicenter data that accounts for regional environmental variations and diverse hospital resource configurations (8–10). Additionally, domestic manufacturing of vacuum blood collection tube raw materials and components lacks unified national standards, and most hospitals in the region have not implemented systematic performance validation for their in-use tubes, exacerbating the quality disparity of collection tubes in clinical practice.

This study aims to systematically evaluate 9 key performance indicators (including appearance, tube strength, stopper shedding, fibrin adhesion, separation gel performance, hemolysis, anticoagulation efficacy, draw volume and result consistency) of common vacuum blood collection tubes used in southwest China, with BD tubes as the reference standard. Its innovations are threefold: first, it adopts a multicenter real-world research design covering 10 institutions across plain and plateau areas, reflecting actual clinical usage scenarios; second, it implements a comprehensive performance validation framework that integrates physical properties, operational safety, and diagnostic result accuracy; third, it aligns the validation indicators with the demand for mutual recognition of test results in Sichuan-Chongqing region. The expected value of this study lies in providing evidence-based guidance for clinical laboratories in tube procurement and establishing a regional quality control paradigm for vacuum blood collection tubes, thereby improving the overall pre-analytical quality of laboratory diagnostics in southwest China.

## 2 Study design

This vacuum blood collection tube verification study was conducted from March to May 2025 across 10 hospitals in Southwest China (8 in Sichuan Province and 2 in Chongqing Municipality) to evaluate the performance and clinical characteristics of disposable vacuum blood collection tubes manufactured by suppliers. All 10 hospitals complied with the basic technical standards for clinical hematology and body fluid testing issued by the National Health Commission of the People’s Republic of China. Ten suppliers provided K_2_EDTA tubes, 3.2% sodium citrate tubes, and serum tubes with or without separation gel as candidate tubes, while BD Vacutainer® K_2_EDTA tubes, 3.2% sodium citrate tubes, and serum tubes were used as control tubes. The verification methods in this study were based on the standard operating procedures of the International Council for Standardization in Haematology (11, 12), the Clinical and Laboratory Standards Institute (13, 14), the Guidelines for Performance Verification of Chinese Vacuum Blood Collection Tubes (WS/T 224–2018), and published literature (15, 16).

### 2.1 Sample collection and processing

The participating medical centers and vacuum blood collection tubes included in this study are summarized in Table 1 and Supplementary Table S1. Consistent with routine clinical laboratory workflows, each hospital designated 2 practicing intermediate-level nurses for blood collection, 1 board-certified intermediate-level laboratory physician for blood analysis, 1 dedicated result recorder, and 4 experienced laboratory staff (part-time) for result consistency verification—all personnel performed their standard clinical duties without additional protocol-specific training to reflect real-world operational conditions.

**Table 1:** Candidate K_2_EDTA, sodium citrate and serum tubes with or without separation gel assessed within the multicentre blood collection tube verification study.

Regions and hospitals	Code	Region type	Altitude (m)	Hospital Tier	Candidate Tube Code	Matched BD Comparator Tube
Dazhou Central Hospital (Si Chuan)	DZ	Plain	310	Tertiary-A	A	BD Vacutainer K_2_EDTA/citrate/serum
Guanghan People’s Hospital (Si Chuan)	GH	Plain	460	Secondary	B	BD Vacutainer K_2_EDTA/citrate/serum
Guangyuan Central Hospital (Si Chuan)	GY	Plain	530	Tertiary-A	C	BD Vacutainer K_2_EDTA/citrate/serum
Ganzi Prefecture People’s Hospital (Si Chuan, High altitude area)	GZ	Plateau	2,560	Tertiary-A	D	BD Vacutainer K_2_EDTA/citrate/serum
Nanchong Central Hospital (Si Chuan)	NC	Plain	280	Tertiary-A	E	BD Vacutainer K_2_EDTA/citrate/serum
Affiliated Hospital of Panzhihua University (Si Chuan)	PZH	Plain	1,100	Tertiary-A	F	BD Vacutainer K_2_EDTA/citrate/serum
Qianjiang District Hospital of Traditional Chinese Medicine (Chong Qing)	QJ	Plain	620	Secondary	G	BD Vacutainer K_2_EDTA/citrate/serum
Sichuan Provincial People’s Hospital (Si Chuan)	SC	Plain	500	Tertiary-A	H	BD Vacutainer K_2_EDTA/citrate/serum
Yibin Second People’s Hospital (Si Chuan)	YB	Plain	330	Secondary	I	BD Vacutainer K_2_EDTA/citrate/serum
Zhongxian People’s Hospital (Chong Qing)	ZX	Plain	220	Secondary	J	BD Vacutainer K2EDTA/citrate/serum

Candidate codes A–J denote distinct commercial brands/manufacturers; the specific production lot number for each brand at each site is detailed in Supplementary Table S1.

As summarized in Table 1, a one-to-one comparison design was adopted for all participating centers: each hospital selected one domestic commercial blood tube brand (labeled A–J) as the candidate tube for each tube subtype (K2EDTA, 3.2% sodium citrate, serum tube with separation gel, serum tube without separation gel), and paired it with the corresponding BD Vacutainer^®^ reference tube of the same tube subtype as the comparator. All candidate tubes and BD control tubes used at each site were assigned unique production lot numbers recorded in Supplementary Table S1 to trace batch-related performance variation. No multiple candidate brands were tested within a single hospital to avoid confounding operational differences from cross-brand parallel testing.

**Figure 1:**
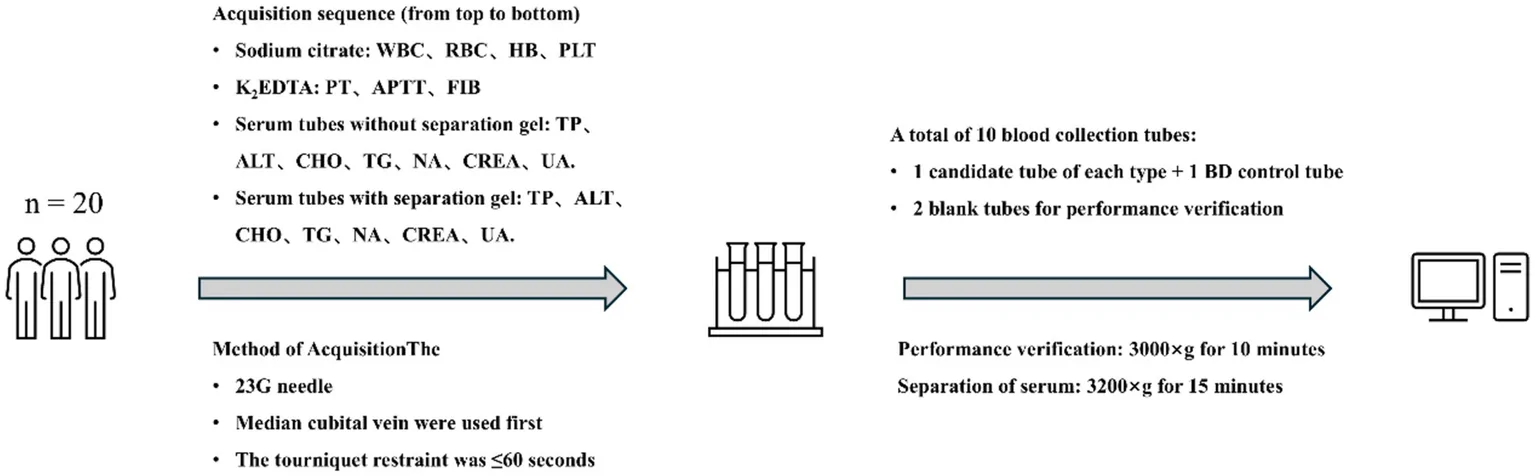
Research flowchart.

A total of 20 adult volunteers (≥18 years old, regardless of gender) were consecutively enrolled per hospital from individuals presenting for routine health check-ups or non-urgent clinical visits, with personal identifiers fully anonymized to protect privacy. Volunteers were excluded only if they had active clinical conditions or were undergoing interventions known to directly invalidate hematological or biochemical test results (i.e., acute viral/bacterial infections requiring systemic antibiotics, ongoing anticoagulant therapy, or pregnancy) were minimized to ensure the study population mirrors real-world users of vacuum blood collection tubes (Figure 1).

This study adhered to the Declaration of Helsinki and the Declaration of Geneva, and all volunteers provided written informed consent prior to enrollment. No additional diagnostic procedures or blood drawings beyond routine clinical practice were performed, ensuring minimal patient burden consistent with real-world research principles (approval number: 2024–224).

Each volunteer donated blood into 10 vacuum collection tubes, with 2–4 mL of blood per tube and a total volume not exceeding 40 mL per individual for the study. All venipuncture operating parameters were fully standardized and uniformly implemented across all 10 participating hospitals to minimize pre-analytical bias.

A uniform 23G blood collection needle was used for all sampling procedures. Venipuncture was primarily performed at the median cubital vein within the antecubital fossa of the upper extremity; only volunteers with unobtainable antecubital venous access underwent sampling via the dorsal hand vein. Tourniquet restraint time was strictly controlled to ≤60 s for each puncture and recorded by nurses on case report forms.

A unified, fixed order of draw consistent with CLSI GP41 guidelines was mandated for all participants and all study centers to prevent additive cross-contamination and sequential sampling bias:

- 3.2% sodium citrate coagulation tubes (blue cap)
- K2EDTA hematology tubes (purple cap)
- Serum tubes without separation gel (red cap)
- Serum tubes with separation gel (yellow cap)

Within each tube category, the sequence of candidate tubes and matched BD comparator tubes was randomly rearranged for every volunteer to balance any residual sequential sampling effects on coagulation, total protein, and biochemical analytes. The same standardized puncture site, needle gauge, and tourniquet timing were maintained throughout collection of all candidate and comparator tubes from a single volunteer, eliminating inter-tube procedural differences. All procedural records including tube draw sequence were archived for subsequent quality verification. All candidate and BD comparator tubes collected from the same volunteer formed matched specimen pairs for subsequent participant-pair clinical consistency analysis.

### 2.2 Performance verification

Prior to blood collection, all candidate tubes and BD comparator control tubes were assigned unique identification numbers for standardized documentation. Each tube was first visually inspected for physical defects in appearance; for serum tubes equipped with separation gel, the homogeneity and integrity of separation gel were assessed as an extra evaluation indicator. For physical performance testing, 30 tubes of each category were randomly selected to draw purified water under actual local environmental conditions. At plain sites, testing was conducted at an ambient pressure of 101 kPa; at plateau sites, the local atmospheric pressure (P_amb) was recorded using a calibrated digital barometer before each test batch (ranging from 74.8 to 85.3 kPa), and tubes were allowed to aspirate water under this authentic working pressure without artificial pressurization, to preserve real-world sampling conditions. The aspirated volume was determined by gravimetry (analytical balance, accuracy 0.001 g), and the mass was converted to volume using the standard density of purified water at 20 °C. This gravimetric approach is the CLSI-recommended reference method for volume verification (CLSI GP34-A2) and remains entirely valid under reduced atmospheric pressure, as it relies on absolute mass measurement rather than pressure-dependent volume expansion. After water aspiration, all tested tubes were placed in the same horizontal centrifuge and spun concurrently at 3000 × g for 10 min to evaluate tube body structural strength. For K2EDTA tubes, 20 specimens that completed fill volume measurement were punctured twice on a hematology analyzer to quantify stopper debris shedding, with the acceptable shedding threshold defined as ≤20%.

For matched candidate and comparator blood specimens collected from the same volunteer for clinical consistency testing, a fully synchronized processing workflow was enforced to eliminate confounding variability from specimen handling, which guarantees any detected analytical differences can be attributed to inherent tube performance rather than inconsistent pre-analytical or analytical operations: paired candidate and BD reference tubes from one participant were loaded side-by-side in the same centrifuge rotor and centrifuged simultaneously with identical speed, duration and temperature parameters specific to each tube subtype; identical pre-centrifugation standing intervals were applied uniformly to paired tubes (60 min vertical rest for serum tubes, immediate inversion mixing followed by 3-h incubation for K2EDTA and sodium citrate tubes) with no differential delay between candidate and comparator groups. All hematology, coagulation and biochemical testing of paired aliquots from a single volunteer was performed on the same site-specific analytical instrument, and matched sample pairs were measured consecutively within one unified analytical run using identical lots of detection reagents, calibrators and internal quality control materials.

### 2.3 Clinical verification

Result consistency of all test tubes was evaluated using common clinical laboratory tests uniformly recognized by the 10 medical centers. All detection reagents and calibrators were handled and stored in accordance with hospital laboratory operating specifications and manufacturer’s instructions. The test items for K_2_EDTA tubes included complete blood count (CBC): White blood cell (WBC), Red blood cell (RBC), Hemoglobin (Hb), and Platelets (PLT). For 3.2% sodium citrate tubes, the test items were coagulation assays: Prothrombin time (PT), Activated partial thromboplastin time (APTT), and Fibrinogen (FIB). For serum tubes with or without separation gel, the test items were blood biochemistry: Total protein (TP), Alanine aminotransferase (ALT), Cholesterol (CHO), Triglyceride (TG), Sodium (NA), Creatinine (CREA), and Uric acid (UA).

### 2.4 Statistical analysis

Excel 2016 (Microsoft Corporation, Redmond, WA, United States), GraphPad Prism (Version 8.0.1, San Diego, CA, United States), and R (Version 4.5.1, Vienna, Austria) were used for all statistical computations. Four hierarchical analytical units were predefined and clearly distinguished for distinct outcome analyses, as detailed below.

All physical-performance indicators (tube appearance, structural strength, stopper shedding, fill-volume compliance, hemolysis rate, fibrin adhesion, separation-gel integrity) were summarized using defective rates, where each individual tested vacuum blood-collection tube served as the observational unit. Physical defect counts and defective proportions were calculated separately for each tube subtype at this level.

Clinical-consistency testing relied on matched specimen pairs: one candidate tube paired with a BD comparator tube of identical tube type collected from the same volunteer. Linear regression, coefficient of determination (R^2^), relative bias (R_b_), and comparison bias (C_b_) were all calculated on these paired samples to eliminate inter-individual biological variation. Specimens with severe hemolysis or spontaneous clotting were excluded from paired comparative analysis. 3.2% sodium-citrate plasma samples were retained exclusively for coagulation-parameter testing and were excluded from complete blood count and biochemical assays.

Defective rates for physical indicators and pass-fail judgments for clinical consistency were reported at the hospital-site level. Stratified site-level results enabled performance comparison between plain-area and plateau-area medical facilities. Valid tube-level and participant-pair datasets from all 10 hospitals were combined to generate pooled summary statistics for characterizing prevalent quality defects of domestic vacuum blood-collection tubes across the diverse terrain of Southwest China.

For pairwise clinical comparison between candidate and BD comparator tubes:

If linear regression yielded R^2^ ≥ 0.95, relative bias was calculated at predefined clinical decision thresholds. If R^2^ < 0.95, comparison bias was computed for every individual paired specimen. A tested tube batch was deemed qualified if either of the following pre-specified criteria was satisfied:

1. *R*^2^ ≥ 0.95 and the relative bias fell within clinically acceptable error limits defined by CLIA’88 and Chinese national health-industry standards;
2. *R*^2^ < 0.95, while ≥80% of all paired-sample comparison-bias values were within acceptable error ranges.

Defective rates were used to describe categorical physical-performance defects at the individual-tube level.

Calculation formulas:

$R_{b}=(B_{x}/X_{c})\times 100\%$ , where *B*_x_ = predicted bias at the medical decision concentration (X_c_);

$C_{b}=[(B_{i2}-B_{i1})/B_{i1}]\times 100\%$ , where *B*_i2_ and *B*_i1_ denote candidate-tube and reference-tube measurement results obtained from the same volunteer, respectively.

## 3 Results

### 3.1 Performance verification

Across all 10 hospitals, K_2_EDTA tubes had purple caps, 3.2% sodium citrate tubes had blue caps, serum tubes with separation gel had yellow caps, and serum tubes without separation gel had red caps. All tubes exhibited tight integration of caps and bodies (no skew, misalignment, or detachment), transparent tube walls, clear graduation marks, and intact labels. No leakage or precipitation of separation gel was observed. Most centers reported significant fill volume errors in 2–3 types of blood collection tubes compared to BD tubes. Among the 10 studies, only the K_2_EDTA tubes in the QJ study met the qualification criteria for shedding rate after two punctures. All tubes passed the tube strength test.

**Figure 2:**
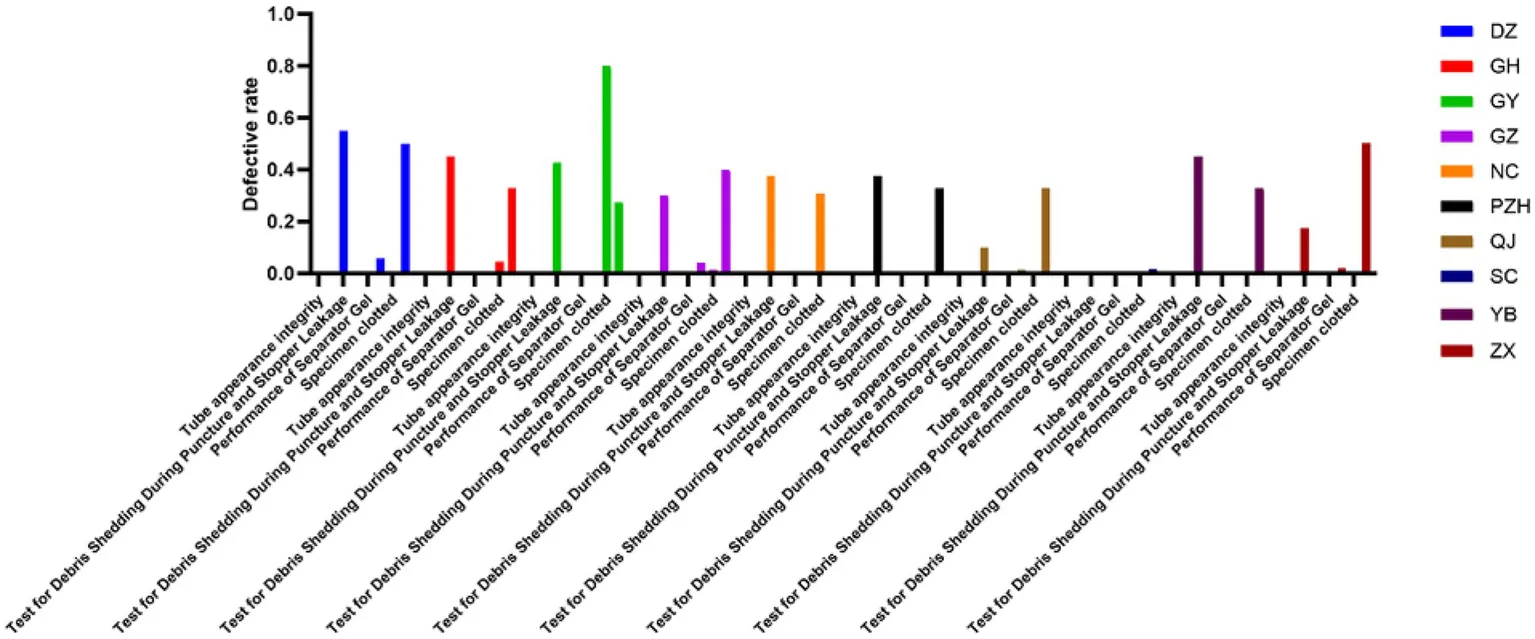
The performance of K2EDTA, Sodium citrate (3.2%), and serum tubes with or without separation gel. DZ, Dazhou Central Hospital; GH, Guanghan People’s Hospital; GY, Guangyuan Central Hospital; GZ, Garzê Prefecture People’s Hospital; NC, Nanchong Central Hospital; PZH, Affiliated Hospital of Panzhihua University; QJ, Qianjiang District Hospital of Traditional Chinese Medicine; SC, Sichuan Provincial People’s Hospital; YB, Yibin Second People’s Hospital; ZX, Zhongxian People’s Hospital.

During blood collection, no fibrin adhesion was observed in any serum tubes, and the serum separation performance of the separation gel was normal. Unacceptable hemolysis rates were detected in non-separating gel serum tubes in the DZ study, 3.2% sodium citrate tubes in the GZ study, and 3.2% sodium citrate tubes in the ZX study; all other tubes showed no hemolysis or acceptable hemolysis rates. Excessive anticoagulation rates were observed in both Candidate tubes and BD’s K_2_EDTA and 3.2% sodium citrate tubes in the GY study, as well as in Candidate K_2_EDTA tubes in the GH study (Figure 2 and Supplementary Table S2).

Supplementary abnormal clotting outcomes were observed for both candidate and BD reference anticoagulant tubes at the GY site: 75% (30/40) of K2EDTA specimens from candidate tubes and 80% (32/40) of matched BD tubes developed microclots; the clotting rate reached 80 and 85% for candidate and BD 3.2% sodium citrate tubes, respectively. Such extremely high clotting proportions across both tube types cannot be attributed to manufacturing defects of collection tubes or insufficient anticoagulant dosage. *Post hoc* review of on-site operating records revealed non-compliant pre-analytical workflows at GY Hospital: phlebotomy staff failed to invert and mix tubes immediately after venipuncture, and specimens were left standing at room temperature for over 2 h before centrifugation due to staffing shortages. This site-specific procedural deviation led to universal microclot formation regardless of tube brand.

### 3.2 Clinical validation: K_2_EDTA and serum tubes

Hematological analysis of K_2_EDTA tubes revealed that the WBC parameter in the GH study failed due to excessive Rb. In the PZH study, Hb (18%) and PLT (11%) parameters were non-compliant due to unacceptable Cb. All K_2_EDTA tube tests in the other studies met the qualification criteria (Table 2).

**Table 2:** Clinical consistency analysis of K_2_EDTA tubes.

Study	WBC		RBC		Hb		PLT	
	*R* ^2^	Decision	*R* ^2^	Decision	*R* ^2^	Decision	*R* ^2^	Decision
DZ	0.9919	Pass	0.9869	Pass	0.9944	Pass	0.9926	Pass
GH	0.9547	No Pass (Rb > Bias)	0.9859	Pass	0.9782	Pass	0.9667	Pass
GY	0.9778	Pass	0.9748	Pass	0.9832	Pass	0.9790	Pass
GZ	0.9748	Pass	0.9917	Pass	0.9929	Pass	0.9709	Pass
NC	0.9686	Pass	0.9839	Pass	0.9882	Pass	0.9647	Pass
PZH	0.9673	Pass	0.9501	Pass	0.9206	No Pass (Cb > Bias)	0.9140	No Pass (Cb > Bias)
QJ	0.9657	Pass	0.9551	Pass	0.9960	Pass	0.9832	Pass
SC	0.9926	Pass	0.9949	Pass	0.9941	Pass	0.9557	Pass
YB	0.9597	Pass	0.9698	Pass	0.9802	Pass	0.9715	Pass
ZX	0.9672	Pass	0.9587	Pass	0.9553	Pass	0.9703	Pass

DZ, Dazhou Central Hospital; GH, Guanghan People’s Hospital; GY, Guangyuan Central Hospital; GZ, Garzê Prefecture People’s Hospital; NC, Nanchong Central Hospital; PZH, Affiliated Hospital of Panzhihua University; QJ, Qianjiang District Hospital of Traditional Chinese Medicine; SC, Sichuan Provincial People’s Hospital; YB, Yibin Second People’s Hospital; ZX, Zhongxian People’s Hospital. WBC, White blood cell; RBC, Red blood cell; Hb, Hemoglobin; PLT, Platelets. Rb, Relative bias.

In the biochemical analysis of serum tubes across all nine studies, the non-compliant parameter based on Cb was serum TP (GH: 16%, GZ: 17%, PZH: 37%, YB: 11%). For hematological tests, the Rb of the WBC parameter in the GH study exceeded the acceptable limit, while the Cb of Hb (18%) and PLT (11%) in the PZH study was non-compliant. And non-compliant Cb was observed for NA in the GZ study and CREA in the NC study. Additionally, unacceptable Rb was found for NA in the SC study. Unacceptable Rb was found for CREA in the GH study and UA in the QJ study. All studies yielded compliant results for ALT, CHO, and TG (Table 3).

**Table 3:** Clinical consistency analysis of serum tubes with or without separation gel.

Study	TP		ALT		CHO		TG		NA		CREA		UA	
	*R* ^2^	Decision	*R* ^2^	Decision	*R* ^2^	Decision	*R* ^2^	Decision	*R* ^2^	Decision	*R* ^2^	Decision	*R* ^2^	Decision
DZ^a^	0.8506	No Pass (Cb > Bias)	0.9815	Pass	0.9929	Pass	0.9998	Pass	0.5641	Pass (Cb < Bias)	0.9738	Pass	0.9978	Pass
GH	0.9235	No Pass (Cb > Bias)	0.9932	Pass	0.9918	Pass	0.9995	Pass	0.7524	Pass	0.9626	No Pass (Rb > Bias)	0.9989	Pass
GY^a^	0.9292	Pass	0.9564	Pass	0.9975	Pass	0.9999	Pass	0.5913	Pass (Cb < Bias)	0.9879	Pass	0.9975	Pass
GZ	0.8403	No Pass (Cb > Bias)	0.9985	Pass	0.9751	Pass	0.9998	Pass	0.5442	No Pass (Cb > Bias)	0.9821	Pass	0.9988	Pass
NC	0.9161	Pass	0.9605	Pass	0.9822	Pass	0.9975	Pass	0.8387	Pass	0.8721	No Pass (60%)	0.9340	Pass
PZH	0.6469	No Pass (Cb > Bias)	0.9958	Pass	0.9625	Pass	0.9991	Pass	0.8009	Pass	0.9704	Pass	0.9969	Pass
QJ	0.9394	Pass	0.9990	Pass	0.9726	Pass	0.9992	Pass	0.7584	Pass	0.9963	Pass	0.9989	No Pass (Rb > Bias)
SC	0.9682	Pass	0.9972	Pass	0.9972	Pass	0.9994	Pass	0.6556	No Pass (Cb > Bias)	0.9707	Pass	0.9718	Pass
YB	0.9192	No Pass (11%)	0.9965	Pass	0.9887	Pass	0.9987	Pass	0.9283	Pass	0.9961	Pass	0.9993	Pass
ZX	0.9588	Pass	0.9962	Pass	0.9933	Pass	0.9992	Pass	0.9542	Pass	0.9771	Pass	0.9986	Pass

DZ, Dazhou Central Hospital; GH, Guanghan People’s Hospital; GY, Guangyuan Central Hospital; GZ, Garzê Prefecture People’s Hospital; NC, Nanchong Central Hospital; PZH, Affiliated Hospital of Panzhihua University; QJ, Qianjiang District Hospital of Traditional Chinese Medicine; SC, Sichuan Provincial People’s Hospital; YB, Yibin Second People’s Hospital; ZX, Zhongxian People’s Hospital. TP, Total protein; ALT, Alanine aminotransferase; CHO, Cholesterol; TG, Triglyceride; K, Potassium; NA, Sodium; CREA, Creatinine; UA, Uric acid. Rb, Relative bias.

^a^For indicators with *R*^2^ < 0.95 but passing Cb reexamination, Bland–Altman difference plots were plotted (Supplementary Figures S1, S2).

### 3.3 Clinical verification: 3.2% sodium citrate tubes

This study conducted a consistency evaluation of the anticoagulant efficacy of 3.2% sodium citrate tubes. For the APTT, relatively high non-compliance rates were observed in the GZ, PZH, and ZX studies (22, 15, and 17%). Notably, significant non-compliance rates were detected for FIB in the GY, QJ, and ZX studies (35, 52, and 40%).

For the PT parameter at the GH site, the *R*^2^ value was 0.4366. According to our predefined criteria, since *R*^2^ was < 0.95, the pass/fail judgment was based on the proportion of individual comparison biases (Cb) within acceptable limits. Re-examination of the paired data revealed that 18 out of 20 (90%) individual Cb values met the acceptability criterion (≥80% threshold), leading to an overall classification of ‘Pass’. The low R^2^ was primarily attributable to two extreme outliers from a single volunteer, which did not represent a systematic analytical bias. For all other parameters initially classified as ‘No Pass’, a similar Cb distribution analysis was performed, confirming that the failures were due to systematic bias (e.g., >20% of Cb values exceeded the limits) (Table 4).

**Table 4:** Clinical consistency analysis of sodium citrate (3.2%) tubes.

Study	PT		APTT		FIB	
	*R* ^2^	Decision	*R* ^2^	Decision	*R* ^2^	Decision
DZ^a^	0.9518	Pass	0.9382	Pass (Cb < Bias)	0.9406	Pass (Cb < Bias)
GH	0.4366	Pass	0.6160	No Pass (Cb > Bias)	0.6335	No Pass (Cb > Bias)
GY^a^	0.8863	Pass	0.8978	Pass (Cb < Bias)	0.9486	No Pass (Cb > Bias)
GZ^a^	0.8259	Pass	0.5790	No Pass (Cb > Bias)	0.8840	Pass (Cb < Bias)
NC	0.8259	Pass	0.9881	Pass	0.9915	Pass
PZH	0.9481	Pass	0.6991	No Pass (Cb > Bias)	0.9172	Pass (Cb < Bias)
QJ^a^	0.7311	Pass	0.5289	Pass (Cb > Bias)	0.9217	No Pass (CRb > Bias)
SC	0.9769	Pass	0.9733	Pass	0.998	Pass
YB^a^	0.9951	Pass	0.9104	Pass (Cb < Bias)	0.9692	Pass
ZX	0.8001	Pass	0.8083	No Pass (Cb > Bias)	0.8898	No Pass (Cb > Bias)

DZ, Dazhou Central Hospital; GH, Guanghan People’s Hospital; GY, Guangyuan Central Hospital; GZ, Garzê Prefecture People’s Hospital; NC, Nanchong Central Hospital; PZH, Affiliated Hospital of Panzhihua University; QJ, Qianjiang District Hospital of Traditional Chinese Medicine; SC, Sichuan Provincial People’s Hospital; YB, Yibin Second People’s Hospital; ZX, Zhongxian People’s Hospital. PT, Prothrombin time; APTT, Activated partial thromboplastin time; FIB, Fibrinogen.

^a^For indicators with *R*^2^ < 0.95 but passing Cb reexamination, Bland–Altman difference plots were plotted (Supplementary Figures S3—S8).

## 4 Discussion

This multicenter real-world study comprehensively evaluated the performance of 10 locally used vacuum blood collection tube brands (specific batches tested between March and May 2025) across tertiary, secondary, and county-level hospitals in Southwest China, covering both plain and plateau areas (e.g., Garzê Prefecture, altitude 2,560 m). Consistent with real-world research principles, the study was conducted under unmodified routine clinical workflows to mirror daily sampling practice, where heterogeneous center-specific factors (equipment, centrifugation, mixing, storage, staff operation) coexist with inherent tube manufacturing defects. The core findings revealed widespread performance deficiencies concentrated on fill volume accuracy and puncture-induced stopper shedding, with fill volume failures markedly aggravated at high altitude. Importantly, all conclusions are restricted to the tested brands, batches, site equipment and 3-month sampling window, and cannot be generalized to all domestic tubes or pediatric/emergency scenarios.

Several real-world mechanisms explain tube-related defects. Insufficient cap sealing and factory QC fluctuation cause fill volume deviation; plateau low atmospheric pressure further disrupt preset vacuum, a pattern confirmed as standard single-wall BD tubes exhibited 100% fill non-compliance in Garzê Prefecture. It is critical to emphasize BD Vacutainer tubes act only as a widely adopted comparative benchmark rather than an error-free absolute gold standard—BD products also display altitude-dependent vacuum defects and identical clotting failure at sites with flawed workflows. Only BD double-walled citrate tubes maintained stable draw volume across elevations, while BD EDTA tubes showed far less stopper debris than domestic alternatives. Excessive stopper shedding in local tubes (100% at DZ, 75% at YB) stems from cost-limited rubber formulations, consistent with prior equipment clogging reports (17).

When contextualized against published work, our multicenter plain-plateau design fills regional research gaps. Overseas controlled trials mainly describe CBC bias under standardized lab settings (18, 19), while most domestic evaluations rely on single-center idealized testing. By contrast, we captured two dominant pre-analytical error sources: intrinsic tube flaws (poor sealing, low-quality stoppers) and variable center workflows (mixing, storage, centrifugation, analyzer/reagent differences), which must be clearly distinguished before attributing abnormal results to tube quality (10, 20). A representative case illustrating workflow-driven interference was the 75–85% microclot rate for both candidate and BD tubes at GY Hospital. Identical failure across all tube types ruled out anticoagulant ratio or vacuum defects; root causes were confirmed as inadequate post-collection inversion and prolonged room-temperature storage. This highlights a core interpretative principle for real-world data: consistent abnormal results across test and comparator tubes at one site originate from non-standardized local operations rather than tube manufacturing. Inter-hospital disparities in fill compliance (0% at NC vs. 100% at ZX) similarly reflect site-specific humidity, storage and phlebotomy technique unrelated to tube batches.

Abnormal PT, APTT and FIB consistency results arise from two separable drivers. Tube-originated coagulation bias mainly stems from underfilling disrupting the fixed 9:1 blood-citrate ratio, uneven anticoagulant coating, or stopper debris activating coagulation cascades. Center-specific confounders include inconsistent inversion, variable pre-spin delay, and inter-site differences in coagulation analyzers/reagent lots. For instance, isolated poor PT correlation at GH was traced to two outlier samples from irregular venipuncture rather than tube defects; elevated APTT bias at the high-altitude GZ site combined citrate tube underfilling (tube factor) and local prolonged specimen holding (center factor); extremely high FIB deviation at QJ corresponded purely to persistent fill volume inaccuracy with no workflow irregularities recorded.

We acknowledge a methodological limitation arising from sample exclusion rules outlined in Methods: hemolyzed and spontaneously clotted specimens were removed from clinical consistency analysis, and citrate tubes were excluded from CBC/biochemistry testing. Hemolysis and microclotting are direct adverse endpoints of defective tube design (unstable vacuum, fragile tube walls, poor stoppers). Filtering these impaired samples before bias calculation inevitably underestimates the full real-world risk of substandard tubes and introduces optimistic pass-rate bias. This exclusion was mandatory to enable uniform cross-center linear regression and bias comparison aligned with WS/T 224-2018, though future dual-analysis designs (one filtered dataset for standardized comparison, one unfiltered cohort to quantify tube-related adverse events) are recommended. We considered mixed-effects modeling to quantitatively partition variance attributable to tube type versus hospital center, but omitted this analysis for two practical reasons: routine regional laboratory batch verification only requires standard linear regression and bias assessment per national guidelines, and the 20-subject per-center sample size lacks sufficient statistical power for stable multi-level variance estimation. Instead, we qualitatively separate tube and workflow effects throughout all stratified site-level result interpretation.

For fill volume non-compliance, the primary contributors include inadequate cap sealing integrity of the tested batches (a common issue in mass-produced local products), factory quality control variations among evaluated brands, and altitude-related atmospheric pressure reduction. Notably, BD’s double-walled blue citrate tubes maintained stable aspiration across elevations, while single-wall BD tubes failed completely at plateau sites. Our data further demonstrates that BD EDTA tubes generate minimal puncture debris, whereas most domestic brands suffered severe stopper shedding linked to low-cost rubber formulations.

The findings of this study hold direct practical value for laboratory management, manufacturing optimization and regional test mutual recognition, confined to our tested product range. Laboratories should implement batch validation prioritizing fill volume and stopper shedding, especially high-altitude facilities. Local tube batches may better adapt to plateau vacuum conditions than standard international single-wall BD products, yet on-site multi-brand validation is still required rather than universal customized production. Manufacturers should upgrade stopper materials and sealing technology to counter altitude pressure shifts (21). This aligns with real-world quality control principles, as routine verification adapts to the inherent variability of mass-produced medical devices. For hospitals in high-altitude areas (≥1,000 m), our data suggest that locally optimized tube batches may offer better fill volume adaptability than standard international brands—but this does not constitute a blanket recommendation for “high-altitude customized production.” Instead, laboratories should conduct on-site validation of multiple brands/batches to match product performance to their specific environmental and equipment conditions. For manufacturers, these real-world data highlight the need for robust cap sealing and vacuum-stabilization technologies to mitigate altitude-induced underfilling. However, we caution against overgeneralizing our single-batch observation. The superior draw-volume performance of one domestic brand at the plateau site does not imply that all domestic tubes are altitude-adapted. Instead, we recommend that high-altitude hospitals implement mandatory batch-specific fill-volume verification prior to procurement, and consider evaluating both domestic and international products side-by-side under their actual environmental conditions (22). Region-wide mutual recognition requires harmonization of both tube procurement standards and core pre-analytical workflows (mixing, storage, centrifugation) to eliminate site-specific confounding (23).

All clinical validations relied on healthy volunteers with narrow analyte concentration ranges, limiting generalizability to patients with extreme abnormal laboratory values. Future work should enroll stratified diseased cohorts to fully verify linearity across clinical measurement intervals.

In conclusion, this real-world multicenter study identifies context-dependent manufacturing defects in domestic vacuum tubes, amplified under high-altitude conditions. Pre-analytical quality improvement demands dual interventions: industrial upgrades targeting fill accuracy and stopper performance, plus standardized cross-hospital specimen handling to mitigate workflow-derived bias. Routine batch verification and site-specific plateau validation will reduce repeat phlebotomy and support reliable inter-hospital test result mutual recognition across Southwest China.

## Data Availability

The original contributions presented in the study are included in the article/Supplementary material, further inquiries can be directed to the corresponding authors.
