# Supplementary material for: Performance evaluation of vacuum blood collection tubes in multicenter clinical laboratories: a real-world study from Southwest China
Source: Front Public Health. 2026 Sep 10;14:1895353. doi: 10.3389/fpubh.2026.1895353 (PMC13601300; doi:10.3389/fpubh.2026.1895353)
Supplement: Supplementary file 1 [file Supplementary_file_1.docx]

**Supplement**

Table S1 Lot Number of Blood Collection Tube

| **Regions and hospitals** | **Code** | **K_2_EDTA** | | **Sodium citrate (3.2%)** | | **Serum tubes with or without separation gel** | |
| --- | --- | --- | --- | --- | --- | --- | --- |
|  |  | **Tube** | **Lot number** | **Tube** | **Lot number** | **Tube** | **Lot number** |
| Dazhou Central Hospital (Si Chuan) | DZ | Candidate Tube A | 202405006 | Candidate Tube A | 20240607 | Candidate Tube A (Without) | 20240921 |
|  |  | Comparator Tube | 4100834 | Comparator Tube | 4113950 | Comparator Tube | 4073301 |
| Guanghan People's Hospital (Si Chuan) | GH | Candidate Tube B | 20240715 | Candidate Tube B | 20240407 | Candidate Tube B (Without) | 20240706 |
|  |  | Comparator Tube | 4100834 | Comparator Tube | 4113950 | Comparator Tube | 4073301 |
| Guangyuan Central Hospital (Si Chuan) | GY | Candidate Tube C | 20240428 | Candidate Tube C | 20240429 | Candidate Tube C (With) | 20240718 |
|  |  | Comparator Tube | 4100834 | Comparator Tube | 4060089 | Comparator Tube | 4082458 |
| Ganzi Prefecture People's Hospital (Si Chuan, High altitude area) | GZ | Candidate Tube D | 24050620 | Candidate Tube D | 24060435 | Candidate Tube D (With) | 48240501 |
|  |  | Comparator Tube | 4100834 | Comparator Tube | 4113950 | Comparator Tube | 4115599 |
| Nanchong Central Hospital (Si Chuan) | NC | Candidate Tube E | 202405160 | Candidate Tube E | 202404150 | Candidate Tube E (Without) | 202402180 |
|  |  | Comparator Tube | 4100834 | Comparator Tube | 4043832 | Comparator Tube | 3274650 |
| Affiliated Hospital of Panzhihua University (Si Chuan) | PZH | Candidate Tube F | 25020715 | Candidate Tube F | 25011624 | Candidate Tube F (With) | 4247513 |
|  |  | Comparator Tube | 4179878 | Comparator Tube | 4267663 | Comparator Tube | 367989 |
| Qianjiang District Hospital of Traditional Chinese Medicine (Chong Qing) | QJ | Candidate Tube G | 02D241103 | Candidate Tube G | 02F241101 | Candidate Tube G (With) | 05C241202 |
|  |  | Comparator Tube | 4179878 | Comparator Tube | 4246057 | Comparator Tube | 4152182 |
| Sichuan Provincial People's Hospital (Si Chuan) | SC | Candidate Tube H | 1016181189 | Candidate Tube H | 101631249 | Candidate Tube H (Without) | 101610843 |
|  |  | Comparator Tube | 367861 | Comparator Tube | 363095 | Comparator Tube | 367812 |
| Yibin Second People's Hospital (Si Chuan) | YB | Candidate Tube I | 2402009A | Candidate Tube I | 2408034A | Candidate Tube I (Without) | 2408067D |
|  |  | Comparator Tube | 4100834 | Comparator Tube | 4113950 | Comparator Tube | 3274650 |
| Zhongxian People's Hospital (Chong Qing) | ZX | Candidate Tube J | 24120201 | Candidate Tube J | 24102801 | Candidate Tube J (Without) | 24101601 |
|  |  | Comparator Tube | 4179878 | Comparator Tube | 4267663 | Comparator Tube | 4094089 |

Table S2. Overview of candidate blood collection tube brands (A–J) and matched BD reference tubes tested at each participating hospital. For every tube category (K2EDTA, 3.2% sodium citrate, serum tubes with/without separation gel), each hospital evaluated one single domestic candidate tube brand against one matched BD comparator tube, with corresponding batch lot numbers listed separately.

| **Tube comparison** | **K_2_EDTA** | | | | **Sodium citrate (3.2%)** | | | | **Serum tubes with separation gel** | | | | **Serum tubes without separation gel** | | | |
| --- | --- | --- | --- | --- | --- | --- | --- | --- | --- | --- | --- | --- | --- | --- | --- | --- |
|  | Candidate Tube | Defective rate | BD | Defective rate | Candidate Tube | Defective rate | BD | Defective rate | Candidate Tube | Defective rate | BD | Defective rate | Candidate Tube | Defective rate | BD | Defective rate |
| **DZ** |  |  |  |  |  |  |  |  |  |  |  |  |  |  |  |  |
| 1.Tube appearance integrity (n=20) | 0 | 0 | 0 | 0 | 0 | 0 | 0 | 0 | — | — | — | — | 0 | 0 | 0 | 0 |
| 2. Strength of tube body (n=30) | 0 | 0 | 0 | 0 | 0 | 0 | 0 | 0 | — | — | — | — | 0 | 0 | 0 | 0 |
| 3. Test for Debris Shedding During Puncture and Stopper Leakage (n=20) | 20 | 100% | 2 | 10% | — | — | — | — | — | — | — | — | — | — | — | — |
| 4. Fibrin adhesion to the tube wall of serum separator tubes (n=40) | — | — | — | — | — | — | — | — | — | — | — | — | 0 | 0 | 0 | 0 |
| 5. Performance of Separator Gel (n=20) | — | — | — | — | — | — | — | — | — | — | — | — | — | — | — | — |
| 6. Haemolysed specimens after centrifugation (n=40) | 0 | 0 | 0 | 0 | 0 | 0 | 0 | 0 | — | — | — | — | 12 | 30% | 2 | 5% |
| 7. Specimen clotted (n=40) | 0 | 0 | 0 | 0 | 0 | 0 | 0 | 0 | — | — | — | — | 0 | 0 | 0 | 0 |
| 8. Draw volume (n=30) | 28 | 93.3% | 0 | 0 | 30 | 100% | 0 | 0 | — | — | — | — | 30% | 100% | 0 | 0 |
| **GH** |  |  |  |  |  |  |  |  |  |  |  |  |  |  |  |  |
| 1. Tube appearance integrity (n=20) | 0 | 0 | 0 | 0 | 0 | 0 | 0 | 0 | — | — | — | — | 0 | 0 | 0 | 0 |
| 2. Strength of tube body (n=30) | 0 | 0 | 0 | 0 | 0 | 0 | 0 | 0 | — | — | — | — | 0 | 0 | 0 | 0 |
| 3. Test for Debris Shedding During Puncture and Stopper Leakage (n=20) | 15 | 75% | 3 | 15% | — | — | — | — | — | — | — | — | — | — | — | — |
| 4. Fibrin adhesion to the tube wall of serum separator tubes (n=40) | — | — | — | — | — | — | — | — | — | — | — | — | 0 | 0 | 0 | 0 |
| 5. Performance of Separator Gel (n=20) | — | — | — | — | — | — | — | — | — | — | — | — | — | — | — | — |
| 6. Haemolysed specimens after centrifugation (n=40) | 0 | 0 | 0 | 0 | 0 | 0 | 0 | 0 | — | — | — | — | 0 | 0 | 0 | 0 |
| 7. Specimen clotted (n=40) | 4 | 10% | 2 | 5% | 1 | 2.50% | 0 | 0 | — | — | — | — | — | — | — | — |
| 8. Draw volume (n=30) | 30 | 100% | 0 | 0 | 4 | 13.3% | 0 | 0 | — | — | — | — | 25 | 83.3% | 0 | 0 |
| **GY** |  |  |  |  |  |  |  |  |  |  |  |  |  |  |  |  |
| 1. Tube appearance integrity (n=20) | 0 | 0 | 0 | 0 | 0 | 0 | 0 | 0 | 0 | 0 | 0 | 0 | — | — | — | — |
| 2. Strength of tube body (n=30) | 0 | 0 | 0 | 0 | 0 | 0 | 0 | 0 | 0 | 0 | 0 | 0 | — | — | — | — |
| 3. Test for Debris Shedding During Puncture and Stopper Leakage (n=20) | 15 | 75% | 2 | 10% | — | — | — | — | — | — | — | — | — | — | — | — |
| 4. Fibrin adhesion to the tube wall of serum separator tubes (n=40) | — | — | — | — | — | — | — | — | 0 | 0 | 0 | 0 | — | — | — | — |
| 5. Performance of Separator Gel (n=20) | — | — | — | — | — | — | — | — | 0 | 0 | 0 | 0 | — | — | — | — |
| 6. Haemolysed specimens after centrifugation (n=40) | 0 | 0 | 0 | 0 | 0 | 0 | 0 | 0 | 0 | 0 | 0 | 0 | — | — | — | — |
| 7. Specimen clotted (n=40) | 30 | 75% | 32 | 80% | 32 | 80% | 34 | 85% | — | — | — | — | — | — | — | — |
| 8. Draw volume (n=30) | 0 | 0 | 0 | 0 | 19 | 63.3% | 0 | 0 | 30 | 100% | 0 | 0 | — | — | — | — |
| **GZ** |  |  |  |  |  |  |  |  |  |  |  |  |  |  |  |  |
| 1. Tube appearance integrity (n=20) | 0 | 0 | 0 | 0 | 0 | 0 | 0 | 0 | 0 | 0 | 0 | 0 | — | — | — | — |
| 2. Strength of tube body (n=30) | 0 | 0 | 0 | 0 | 0 | 0 | 0 | 0 | 0 | 0 | 0 | 0 | — | — | — | — |
| 3. Test for Debris Shedding During Puncture and Stopper Leakage (n=20) | 9 | 45% | 3 | 15% | — | — | — | — | — | — | — | — | — | — | — | — |
| 4. Fibrin adhesion to the tube wall of serum separator tubes (n=40) | — | — | — | — | — | — | — | — | 0 | 0 | 0 | 0 | — | — | — | — |
| 5. Performance of Separator Gel (n=20) | — | — | — | — | — | — | — | — | 0 | 0 | 0 | 0 | — | — | — | — |
| 6. Haemolysed specimens after centrifugation (n=40) | 0 | 0 | 2 | 0 | 9 | 25% | 0 | 0 | 0 | 0 | 0 | 0 | — | — | — | — |
| 7. Specimen clotted (n=40) | 2 | 5% | 0 | 0 | 0 | 0 | 0 | 0 | — | — | — | — | — | — | — | — |
| 8. Draw volume (n=30) | 9 | 30% | 30 | 100% | 1 | 3% | 0 | 0 | 2 | 6.7% | 30 | 100% | — | — | — | — |
| **NC** |  |  |  |  |  |  |  |  |  |  |  |  |  |  |  |  |
| 1. Tube appearance integrity (n=20) | 0 | 0 | 0 | 0 | 0 | 0 | 0 | 0 | — | — | — | — | 0 | 0 | 0 | 0 |
| 2. Strength of tube body (n=30) | 0 | 0 | 0 | 0 | 0 | 0 | 0 | 0 | — | — | — | — | 0 | 0 | 0 | 0 |
| 3. Test for Debris Shedding During Puncture and Stopper Leakage (n=20) | 12 | 60% | 3 | 15% | — | — | — | — | — | — | — | — | — | — | — | — |
| 4. Fibrin adhesion to the tube wall of serum separator tubes (n=40) | — | — | — | — | — | — | — | — | — | — | — | — | 0 | 0 | 0 | 0 |
| 5. Performance of Separator Gel (n=20) | — | — | — | — | — | — | — | — | — | — | — | — | — | — | — | — |
| 6. Haemolysed specimens after centrifugation (n=40) | 0 | 0 | 0 | 0 | 0 | 0 | 0 | 0 | — | — | — | — | 0 | 0 | 0 | 0 |
| 7. Specimen clotted (n=40) | 0 | 0 | 0 | 0 | 0 | 0 | 0 | 0 | — | — | — | — | 0 | 0 | 0 | 0 |
| 8. Draw volume (n=30) | 30 | 100% | 0 | 0 | 0 | 0 | 0 | 0 | — | — | — | — | 25 | 83.3% | 0 | 0 |
| **PZH** |  |  |  |  |  |  |  |  |  |  |  |  |  |  |  |  |
| 1. Tube appearance integrity (n=20) | 0 | 0 | 0 | 0 | 0 | 0 | 0 | 0 | 0 | 0 | 0 | 0 | — | — | — | — |
| 2. Strength of tube body (n=30) | 0 | 0 | 0 | 0 | 0 | 0 | 0 | 0 | 0 | 0 | 0 | 0 | — | — | — | — |
| 3. Test for Debris Shedding During Puncture (n=20) | 14 | 70% | 1 | 5% | — | — | — | — | — | — | — | — | — | — | — | — |
| 4. Fibrin adhesion to the tube wall of serum separator tubes (n=40) | — | — | — | — | — | — | — | — | 0 | 0 | 0 | 0 | — | — | — | — |
| 5. Performance of Separator Gel (n=20) | — | — | — | — | — | — | — | — | 0 | 0 | 0 | 0 | — | — | — | — |
| 6. Haemolysed specimens after centrifugation (n=40) | 0 | 0 | 0 | 0 | 0 | 0 | 0 | 0 | 0 | 0 | 0 | 0 | — | — | — | — |
| 7. Specimen clotted (n=40) | 0 | 0 | 0 | 0 | 0 | 0 | 0 | 0 | 0 | 0 | 0 | 0 | — | — | — | — |
| 8. Draw volume (n=30) | 30 | 100% | 0 | 0 | 0 | 0 | 0 | 0 | 0 | 0 | 0 | 0 | — | — | — | — |
| **QJ** |  |  |  |  |  |  |  |  |  |  |  |  |  |  |  |  |
| 1. Tube appearance integrity (n=20) | 0 | 0 | 0 | 0 | 0 | 0 | 0 | 0 | 0 | 0 | 0 | 0 | — | — | — | — |
| 2. Strength of tube body (n=30) | 0 | 0 | 0 | 0 | 0 | 0 | 0 | 0 | 0 | 0 | 0 | 0 | — | — | — | — |
| 3. Test for Debris Shedding During Puncture and Stopper Leakage (n=20) | 3 | 15% | 1 | 5% | — | — | — | — | — | — | — | — | — | — | — | — |
| 4. Fibrin adhesion to the tube wall of serum separator tubes (n=40) | — | — | — | — | — | — | — | — | 0 | 0 | 0 | 0 | — | — | — | — |
| 5. Performance of Separator Gel (n=20) | — | — | — | — | — | — | — | — | 0 | 0 | 0 | 0 | — | — | — | — |
| 6. Haemolysed specimens after centrifugation (n=40) | 0 | 0 | 0 | 0 | 2 | 5% | 0 | 0 | 1 | 2.50% | 0 | 0 | — | — | — | — |
| 7. Specimen clotted (n=40) | 0 | 0 | 0 | 0 | 0 | 0 | 0 | 0 | 0 | 0 | 0 | 0 | — | — | — | — |
| 8. Draw volume (n=30) | 30 | 100% | 0 | 0 | 29 | 96.7% | 0 | 0 | 0 | 0 | 0 | 0 | — | — | — | — |
| **SC** |  |  |  |  |  |  |  |  |  |  |  |  |  |  |  |  |
| 1. Tube appearance integrity (n=20) | 0 | 0 | 0 | 0 | 0 | 0 | 0 | 0 | — | — | — | — | 0 | 0 | 0 | 0 |
| 2. Strength of tube body (n=30) | 0 | 0 | 0 | 0 | 0 | 0 | 0 | 0 | — | — | — | — | 0 | 0 | 0 | 0 |
| 3. Test for Debris Shedding During Puncture and Stopper Leakage (n=20) | 0 | 0 | 0 | 0 | 0 | 0 | 0 | 0 | — | — | — | — | 0 | 0 | 0 | 0 |
| 4. Fibrin adhesion to the tube wall of serum separator tubes (n=40) | 0 | 0 | 0 | 0 | 0 | 0 | 0 | 0 | — | — | — | — | 0 | 0 | 0 | 0 |
| 5. Performance of Separator Gel (n=20) | — | — | — | — | — | — | — | — | — | — | — | — | — | — | — | — |
| 6. Haemolysed specimens after centrifugation (n=40) | 0 | 0 | 0 | 0 | 0 | 0 | 0 | 0 | — | — | — | — | 0 | 0 | 0 | 0 |
| 7. Specimen clotted (n=40) | 0 | 0 | 0 | 0 | 0 | 0 | 0 | 0 | — | — | — | — | 0 | 0 | 0 | 0 |
| 8. Draw volume (n=30) | 3 | 10% | 0 | 0 | 0 | 0 | 0 | 0 | — | — | — | — | 0 | 0 | 0 | 0 |
| **YB** |  |  |  |  |  |  |  |  |  |  |  |  |  |  |  |  |
| 1. Tube appearance integrity (n=20) | 0 | 0 | 0 | 0 | 0 | 0 | 0 | 0 | — | — | — | — | 0 | 0 | 0 | 0 |
| 2. Strength of tube body (n=30) | 0 | 0 | 0 | 0 | 0 | 0 | 0 | 0 | — | — | — | — | 0 | 0 | 0 | 0 |
| 3. Test for Debris Shedding During Puncture and Stopper Leakage (n=20) | 15 | 75% | 3 | 15% | — | — | — | — | — | — | — | — | — | — | — | — |
| 4. Fibrin adhesion to the tube wall of serum separator tubes (n=40) | — | — | — | — | — | — | — | — | — | — | — | — | 0 | 0 | 0 | 0 |
| 5. Performance of Separator Gel (n=20) | — | — | — | — | — | — | — | — | — | — | — | — | — | — | — | — |
| 6. Haemolysed specimens after centrifugation (n=40) | 0 | 0 | 0 | 0 | 0 | 0 | 0 | 0 | — | — | — | — | 0 | 0 | 0 | 0 |
| 7. Specimen clotted (n=40) | 0 | 0 | 0 | 0 | 0 | 0 | 0 | 0 | — | — | — | — | 0 | 0 | 0 | 0 |
| 8. Draw volume (n=30) | 30 | 100% | 0 | 0 | 29 | 96.7% | 0 | 0 | — | — | — | — | 0 | 0 | 0 | 0 |
| **ZX** |  |  |  |  |  |  |  |  |  |  |  |  |  |  |  |  |
| 1. Tube appearance integrity (n=20) | 0 | 0 | 0 | 0 | 0 | 0 | 0 | 0 | — | — | — | — | 0 | 0 | 0 | 0 |
| 2. Strength of tube body (n=30) | 0 | 0 | 0 | 0 | 0 | 0 | 0 | 0 | — | — | — | — | 0 | 0 | 0 | 0 |
| 3. Test for Debris Shedding During Puncture and Stopper Leakage (n=20) | 5 | 25% | 2 | 10% | — | — | — | — | — | — | — | — | — | — | — | — |
| 4. Fibrin adhesion to the tube wall of serum separator tubes (n=40) | — | — | — | — | — | — | — | — | — | — | — | — | 0 | 0 | 0 | 0 |
| 5. Performance of Separator Gel (n=20) | — | — | — | — | — | — | — | — | — | — | — | — | — | — | — | — |
| 6. Haemolysed specimens after centrifugation (n=40) | 1 | 2.50% | 0 | 0 | 2 | 5% | 0 | 0 | — | — | — | — | 1 | 2.50% | 1 | 2.50% |
| 7. Specimen clotted (n=40) | 0 | 0 | 0 | 0 | 0 | 0 | 0 | 0 | — | — | — | — | 0 | 0 | 0 | 0 |
| 8. Draw volume (n=30) | 30 | 100% | 0 | 0 | 30 | 100% | 0 | 0 | — | — | — | — | 30 | 100% | 0 | 0 |

DZ, Dazhou Central Hospital; GH, Guanghan People's Hospital; GY, Guangyuan Central Hospital; GZ, Garzê Prefecture People's Hospital; NC, Nanchong Central Hospital; PZH, Affiliated Hospital of Panzhihua University; QJ, Qianjiang District Hospital of Traditional Chinese Medicine; SC, Sichuan Provincial People's Hospital; YB, Yibin Second People's Hospital; ZX, Zhongxian People's Hospital.

Footnote 1: Each hospital was allocated a unique domestic candidate tube brand (A–J). Within one hospital, only one candidate brand was tested for all tube subtypes, paired with subtype-matched BD comparator tubes with independent lot numbers.

Figure S1 Bland-Altman plot of NA in Dazhou Central Hospital. Range of intercomparison deviation: -2~2%

Figure S2 Bland-Altman plot of NA in Guangyuan Central Hospital. Range of intercomparison deviation: -2~2%

Figure S3 Bland-Altman plot of APTT in Dazhou Central Hospital. Range of intercomparison deviation: -7.5~7.5%

Figure S4 Bland-Altman plot of FIB in Dazhou Central Hospital. Range of intercomparison deviation: -10~10%

Figure S5 Bland-Altman plot of APTT in Guangyuan Central Hospital. Range of intercomparison deviation: -7.5~7.5%

Figure S6 Bland-Altman plot of FIB in Garzê Prefecture People's Hospital. Range of intercomparison deviation: -10~10%

Figure S7 Bland-Altman plot of APTT in Qianjiang District Hospital of Traditional Chinese Medicine. Range of intercomparison deviation: -7.5~7.5%

Figure S8 Bland-Altman plot of APTT in Yibin Second People's Hospital. Range of intercomparison deviation: -7.5~7.5%
